# Effect of 2,4-Thiazolidinedione on Limousin Cattle Growth and on Muscle and Adipose Tissue Metabolism

**DOI:** 10.1155/2012/891841

**Published:** 2012-12-06

**Authors:** M. Arévalo-Turrubiarte, L. González-Dávalos, A. Yabuta, J. D. Garza, J. L. Dávalos, O. Mora, A. Shimada

**Affiliations:** ^1^Programa de Posgrado en Ciencias de la Producción y de la Salud Animal, Universidad Nacional Autónoma de México (UNAM), 54714 Mexico City, DF, Mexico; ^2^Laboratorio de Rumiología y Metabolismo Nutricional (RuMeN), Facultad de Estudios Superiores Cuautitlán (FES Cuautitlán), UNAM, Bulevar B. Quintana 514-D, Colonia Arboledas, 75230 Querétaro, QRO, Mexico; ^3^Facultad de Medicina Veterinaria y Zootecnia (FMVZ), UNAM and Centro de Enseñanza, Investigación y Extensión en Producción Animal en el Altiplano (CEIEPAA), 76790 Tequisquiapan, QRO, Mexico

## Abstract

The main adipogenic transcription factor PPAR*γ* possesses high affinity to 2,4-TZD, a member of the Thiazolidinedione family of insulin-sensitizing compounds used as adipogenic agents. We evaluated 2,4-TZD's effect on bovine growth and PPAR tissue expression. Seventeen Limousin bulls (18 month-old; 350 kg body weight (BW)) were assigned into 2 treatments: control and 2,4-TZD (8 mg/70 kg BW) and were fed until bulls reached 500 kg BW. They were weighed and their blood was sampled. DNA, RNA, and protein were determined in liver; skeletal muscle; subcutaneous (SC), omental, perirenal adipose tissues (AT) to determine protein synthesis rate and cellular size. Expression of PPAR mRNA was measured in liver and muscle (PPAR*α*, -*δ*, and -*γ*) and SC adipose tissue (*γ*) by real-time PCR. No significant differences were found (*P* > 0.1) in weight gain, days on feed, and carcass quality. Muscle synthesis was greater in controls (*P* < 0.05); cell size was larger with 2,4-TZD (*P* < 0.05). PPAR*α*, -*δ*, and -*γ* expressions with 2,4-TZD in liver were lower (*P* < 0.01) than in muscle. No differences were found for PPAR**γ** mRNA expression in SCAT. The results suggest the potential use of 2,4-TZD in beef cattle diets, because it improves AT differentiation, liver, and muscle fatty acid oxidation that, therefore, might improve energy efficiency.

## 1. Introduction

Peroxisome proliferator-activated receptors (PPAR) are ligand-activated transcription factors that belong to the nuclear hormone receptor superfamily. Three isotypes have been identified in lower vertebrates and mammals: PPAR*α* or NR1C1; PPAR*β*/*δ* or NR1C2, also called NUC-1 or FAAR; PPAR*γ* or NR1C3. These receptors exhibit different tissue distribution and functions and, to some extent, different ligand specificities. Mechanistically, they form heterodimers with the retinoid X receptor (RXR) and activate transcription by binding to a specific DNA element, termed the peroxisome proliferator response element (PPRE), in the regulatory region of a variety of genes encoding proteins that are involved in lipid metabolism [1] and energy balance [2–4]. The Thiazolidinediones (TZDs) are PPAR*γ* agonists that possess clinical antidiabetic efficacy, mainly through their actions in adipose tissues [3]. The influence of TZD on adipose differentiation has been demonstrated through modifications on adipose tissue deposition and increases of intramuscular (IM) fat in meat animals [5, 6]; on the other hand, Michalik et al. [3] mentioned that some TZD may act on different PPAR, especially PPAR*α*.

Marbling or IM fat has been positively correlated with meat quality [7] because of the improvement in beef tenderness and palatability [8]. The development of IM fat primarily depends on the animal's breed, gender, age, and nutrition [9, 10]. Therefore, adipose tissue and its metabolic regulation have been studied in order to improve meat characteristics during fattening in feedlots.

The observations suggest that the quality and value of beef cattle carcasses could be increased through the utilization of these compounds as promoters of marbling and that, additionally, they might improve their energy efficiency.

## 2. Materials and Methods

### 2.1. Animals, Treatment, and Diet

Procedures involving animals were approved by the Institutional Committee for Experimental Animal Care of the Universidad Nacional Autónoma de México (UNAM) [11]. Eight bulls served as Controls (Cs) and nine as the treated group (T), which were fed 2,4-TZD (8 mg/70 kg BW); the dose was adjusted to the animal's total weight; this was according to previous pharmacological and clinical studies in humans treated by oral administration (rosiglitazone 8 mg/day), which demonstrated important changes in the metabolism of glucose during insulin resistance treatments, due to its impact on muscular and adipose tissues [12, 13]. In order to test 2,4-TZD by a practical method, we decided to use oral administration as given to humans and mice; to date, this is the first study to employ it within the food in ruminants; other studies have used intravenous administration [14]. Animals were assigned to treatments in a completely randomized design and housed pairwise on dirt-floor pens. Prior to the initiation of the experiment, they were immunized against the bovine respiratory disease complex (BRDC), the bovine viral diarrhea virus (BVDV), and leptospirosis (Cattlemaster 4; Pfizer Animal Health, Exton, PA, USA), injected with vitamins A, D, and E (Vigantol ADE; Merck KgaA, Darmstadt, Germany), implanted with 140 mg trenbolone acetate and 20 mg 17 beta estradiol (Revalor; Intervet/Schering-Plough Animal Health), and protected against ectoparasites (Tiguvon Spot-on, Bayer, Germany). Animals with >90 days of participation in the experiment were reimplanted and reinjected with the vitamins. The diet consisted in forage (alfalfa hay 80% Dry matter (DM)) and a supplement (89% DM, with 15% CP; 2.8 Mcal ED/kg DM), at a 2 : 1 relationship, respectively. The amount offered was based on 3% of BW (ENm: 1.5 Mcal/kg; ENg: 0.9 Mcal/kg, 14.9% protein). Feed was provided twice a day. Treated animals (T) received 2,4-Thiazolidinedione 90% (2,4-TZD) (Sigma Aldrich, St. Louis, MO, USA) in a dose of 8 mg/70 kg BW per animal [12] mixed within the supplement. Animals were individually weighed and their blood was sampled from the coccygeal vein (10 mL) at the 28-day intervals throughout the 196-day study; blood collection and weight were taken before receiving their first meal (on fasting) on the sampling day. Samples were collected in Vacutainer tubes (Hunan, China) without anticoagulants. They were centrifuged (at 13,000 rpm) and the serum was recovered and frozen at −70°C until analysis.

### 2.2. Slaughter, Sampling, and Analysis

As animals reached 500 kg of weight, C and T animals were sent to a nearby federal inspection-type abattoir (TIF) operated under Mexican federal inspection laws [15, 16]. Samples of liver, muscle, and adipose tissue from omental, perirenal, and SC depots were dissected, collected in CryoTubes (Nunc Cryo Tube vials; Roskilde, Denmark), frozen in liquid nitrogen, and stored at −70°C for subsequent analysis. Carcasses were chilled at −2°C for 32–36 h and quality and yield grades were evaluated, with assessment including the following: external fat thickness; *longissimus dorsi* muscle (LM) area at 7th and 8th rib; meat and fat color; marbling, according to North American Meat Processors Association (NAMPA) [17] standards.

### 2.3. Total RNA and DNA Isolation

Total RNAs from all tissue samples (100 mg each) were purified using 1 mL of TRIzol reagent (Invitrogen, Carlsbad, CA, USA) according to the manufacturer's instructions. RNA was eluted in 40 *μ*L of diethylpyrocarbonate- (DEPC-) treated water and quantified by spectrophotometer; samples were then stored at −70°C until further molecular biology experiments. DNA extraction (1 mL/50 mg of tissue) of DNAzol reagent (Invitrogen) was made according to the manufacturer's protocol and eluted in 50 *μ*L of 8 mM sodium hydroxide (NaOH). Spectrophotometry reading was performed (Nano Drop 1000; Thermo Fisher Scientific, Inc., Wilmington, DE, USA) and samples were stored at −20°C. RNA integrity was verified by agarose gel electrophoresis (Seakem LE Agarose, Rockland, ME, USA).

### 2.4. Total Protein Isolation

Protein was extracted from tissues according to Garcia and Phillips [18]. The extraction solution contained the following: 1 M (Tris mol L^−1^), 0.1 M EDTA (mol L^−1^), 1 M sodium chloride (NaCl), 0.1% protease inhibitor cocktail; 0.5% sodium azide (NaN_3_). A 500 *μ*L extraction solution plus 50 mg tissue was incubated overnight at 5°C under agitation. Subsequent centrifugation at 13,000 rmp for 10 min was performed to obtain total protein, which was spectrophotometrically determined by Bradford's assay [19] (Hewlett Packard Agilent 8453 UV-visible (595 nm)) with an albumin standard curve.

### 2.5. Extraction of 2,4-TZD from Tissues

Extractions of 2,4-TZD from tissue samples (250 mg) were carried out using 500 *μ*L of 20% KOH in methanol at 65°C for 45 min; then, 2 mL of diethyl ether were added and two washings of an equal volume of water were performed to remove KOH. The upper phase was evaporated under nitrogen atmosphere; residues were dissolved in the mobile phase of ammonium acetate-acetonitrile and transferred into vials [20].

### 2.6. Extraction of 2,4-TZD from a Concentrate

To ensure the presence of 2,4-TZD in the concentrate feed, we conducted a high-performance liquid chromatography (HPLC) analysis. Five grams were mixed with 7.5 mL of extraction solution (hexane : acetone : alcohol : toluene, 10 : 7 : 6 : 7) in a 25 mL volumetric flask, hand shaken for 1 min, and under conditions of darkness overnight (16 h). Subsequently, KOH (0.5 mL) at 40% was added, shaken, filled with Na_2_SO_4_ at 10%, and maintained again in the dark for 1 h. The extract (7 mL) was placed into Falcon tubes for further nitrogen evaporation. The residue was dissolved in the mobile phase of ammonium acetate-acetonitrile and was transferred in vials to be analyzed by HPLC [20].

### 2.7. Determination of 2,4-TZD by High-Performance Liquid Chromatographic Method (HPLC)

Chromatography for separation and determination of 2,4-TZD was carried out on an HPLC 1046A (Hewlett Packard) with a fluorescence detector. Separation and determination were performed utilizing a 5 *μ*m C18 column (250 × 4.6 mm) (Phenomenex, Torrance, CA, USA) [21]. A reference standard of 2,4-TZD was used (Sigma). The mobile phase consisted of ammonium acetate (Fisher Scientific Company, Fairlawn, NJ, USA) 0.01 M in acetonitrile HPLC grade reagent (J.T. Baker, SOLUSORB; Mallinckrodt Baker, Inc., Paris, KY, USA), and the pH was adjusted to 8.0 at a ratio of 65 : 35 v/v, as described by Muxlow et al. [22]. A stock solution of 2,4-TZD was prepared at a concentration of 1 mg/mL of the mobile phase as diluent. Plasma samples (500 *μ*L) were prepared according to He et al. [23]; these were diluted with 500 *μ*L of acetonitrile and the mixture was agitated in vortex for 3 min and centrifuged at 13,000 rpm for 10 min. The upper phase was evaporated under nitrogen atmosphere. This was reconstituted with the mobile phase and transferred onto a conical insert in a vial with insert (100 *μ*L) (amber autosampler vials; Agilent Technologies, Santa Clara, CA, USA). Chromatographic separations were performed at room temperature at a flow rate of 0.7 mL/min with a fluorescence detector at a 269 nm wavelength. Injections were made by a duplicate with 50 *μ*L of sample.

### 2.8. Quantification of PPAR by Real-Time PCR

In order to quantify PPAR expression in liver, muscle, and SC adipose tissue, cDNA was isolated from the total RNA using oligo (dT)_12–18_ (Sigma) primer and SuperScript II Reverse transcriptase (RT) (Invitrogen) following the manufacturer's instructions. PPAR*α* primers were the following: forward 5′-AGCCTCTGGCTACCACTACG, reverse 5′-CATCCCAACTGAAAGGCACT-3′; PPAR*δ* forward 5′-GGTGACCCTGCTCAAGTACG-3′, reverse 5′-ACTTGACGGCAAACTCGAAC-3′; PPAR*γ* forward 5′-CCATCATGAAGTGTGACGTTG-3′, reverse 5′-ACAGAGTACTTGCGCTCAGGA-3′-PPIA was employed as a housekeeping gene: forward 5′-AGCACTGGGGAGAAAGGATT-3′, reverse 5′-AGCCACTCAGTCTTGGCAGT-3′.

Samples were analyzed in a LightCycler (1.5 instrument; Roche Diagnostics, Basel, Switzerland); a LightCycler FastStart DNA Master Sybr Green I (Roche) was used as well as Capillary Formulation (Sigma). PCR conditions for *PPARα* and *PPIA* genes were as follows: initial denaturation, 95°C/10 min; second denaturation, 95°C/10 sec; annealing, 56°C/10 sec; amplification, 72°C/10 sec for 55 cycles. PPAR*δ* conditions were as follows: denaturation, 95°C/10 min; second denaturation, 95°C/10 sec; annealing, 56°C/10 sec; amplification 72°C/8 sec, for 55 cycles, and for PPAR*γ*, denaturation, 95°C/10 min; second denaturation, 95°C/10 sec; annealing, 58°C/10 sec; amplification, 72°C/8 sec, for 55 cycles. Samples were analyzed by the duplicate; to determine a relative expression of the target genes, they were compared with the reference gene employing the 2^−ΔCt^ method.

## 3. Statistical Analysis

Animals were sorted by weight in a completely randomized design with two treatments (Control C versus 2,4-TZD, T) and four replicates. Carcass yield, *LM* rib-eye area, and fat thickness were compared with Duncan's test (*P* < 0.05); DWG, days on feed, and initial and final liveweight were tested by a linear regression analysis. Synthesis and cellular size were analyzed with the least squares means (LS MEANS) procedure. All data were analyzed with SAS system statistical software package [24].

## 4. Results

### 4.1. Growth Performance

No differences were found (*P* > 0.05) between treatments in DWG, days on feed, initial and final liveweight, carcass yield, *LM* rib-eye area, or fat thickness (Table 1).

### 4.2. Carcass Quality

Carcass evaluation was according to NAMPA [17] procedures. Fat and meat color, carcass morphology, and marbling data showed no differences between treatments. Fat coloration observed in both treatments, according to the Pantone colorimetric system, was 7499, which represents cream coloration. Meat color was reported as 1805C in both treatments; according to quality standards in meat coloration, this tonality is reported in selected and standard carcasses. Marbling in *LM* resulted in nongrade carcasses, which indicated no traces of fat in either treatments (Table 1).

### 4.3. Total DNA, RNA, and Protein in Tissues

To observe the effect of 2,4-TZD on metabolically related tissues, cellular synthesis and size were measured. Cellular synthesis was estimated with RNA/DNA ratios, and cellular size with DNA/protein ratios (results are shown in Table 2).

No differences in hepatic cellular synthesis (*P* = 0.135) and size (*P* = 0.090) were exhibited between treatments. Muscle cell synthesis was lower (*P* < 0.05) in animals treated with 2,4-TDZ, contrary to cell size, which was higher as compared with (C) animals.

Visceral adipose tissue (omentum and perirenal) and SC tissue were sampled to compare adipose depots. Omentum only showed differences (*P* < 0.001) in cell synthesis between (T) and (C). Perirenal samples (*P* < 0.05) had lower cell synthesis in (T) (Table 2), while (T) animals were the largest in size. SC tissue resulted in higher synthesis (*P* < 0.0001) in treatment than in control tissues, contrary to cell size (*P* < 0.01), which was smaller for (T).

### 4.4. Concentration of 2,4-TZD in Blood, Tissues, and Supplement

No peak was detected in the blood of either treatments when compared with the internal standard, demonstrating the absence of compounds related with 2,4-TZD metabolism, as was expected since plasmatic clearance in humans treated with TZDs occurs after 3-4 hours of administration [12]. The 2,4-TZD residue in hepatic tissue was significantly (*P* < 0.0001) different between the treatments (Table 3). No peaks regarding the presence of 2,4-TZD residue in (T) muscle sample analysis appeared from HPLC analyses.

### 4.5. PPAR Expression in Tissues

**Figure 1:**
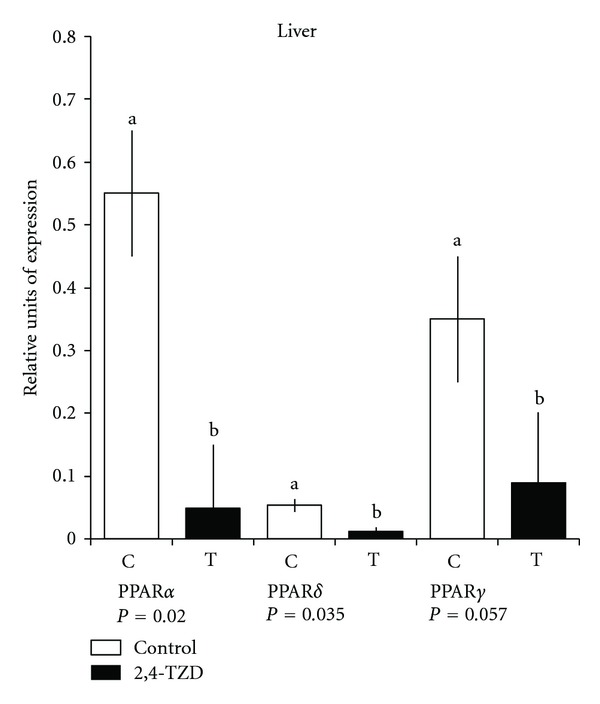
Quantitative polymerase chain reaction (qPCR) of peroxisome proliferation-activated receptor (PPAR)*α*, -*δ*, and -*γ* in the liver. Columns show relative expression of PPAR (*α*, *δ*, and *γ*) in Control (C) and Thiazolidinedione (TZD) (T) groups. ^a,b^Different lowercase letters indicate a significant difference.

**Figure 2:**
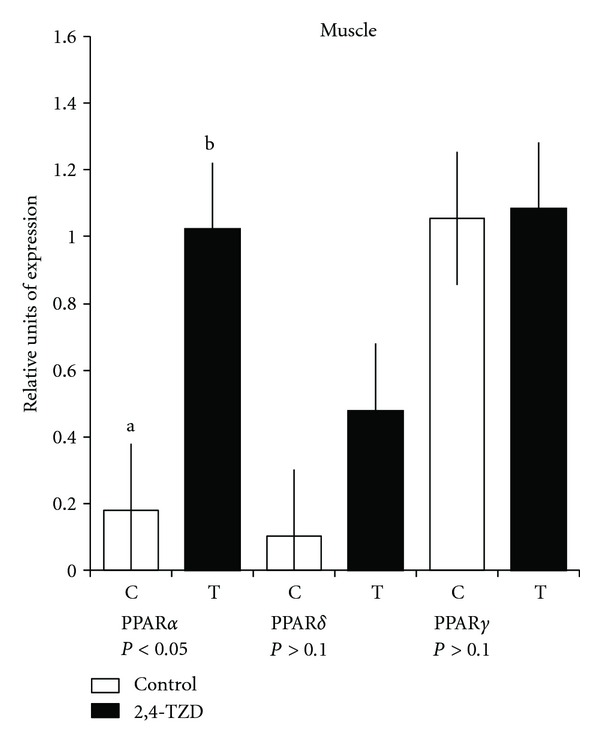
Quantitative polymerase chain reaction (qPCR) of peroxisome proliferation-activated receptors (PPAR)*α*, -*δ*, and -*γ* in the muscle. Columns show relative expression of PPAR (*α*, *δ*, and *γ*) in Control (C) and Thiazolidinedione (TDZ) (T) groups. ^a,b^Different lowercase letters indicate a significant difference.

**Figure 3:**
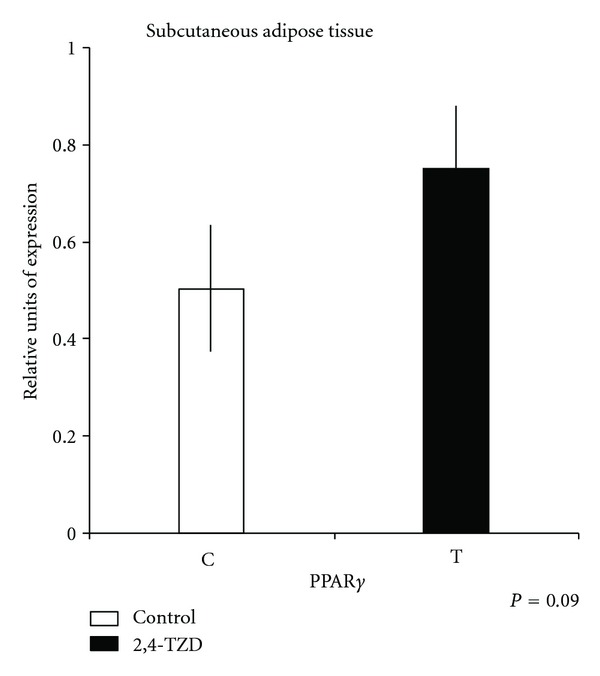
Quantitative polymerase chain reaction (qPCR) of peroxisome proliferation-activated receptor (PPAR)*γ* in subcutaneous (SC) adipose tissue. Columns show relative expression of PPAR*γ* in Control (C) and Thiazolidinedione (TDZ) (T) groups.

Expressions of PPAR in liver (Figure 1) were significantly different between treatments (*P* < 0.01); all PPAR exhibited greater expression in Control (C) animals than in treated (T) ones. In the case of muscle, only PPAR*α* was different (*P* < 0.05) between treatments (Figure 2). There was no difference in PPAR*γ* between treatments (*P* > 0.1) (Figure 3).

## 5. Discussion

### 5.1. Growth Performance

Thiazolidinediones are widely used antidiabetic drugs with proven efficacy mainly as surrogate markers of diabetes management. However, the latter may not always translate into benefits in clinical outcomes. In humans, common side effects associated with TZD include an average weight gain of 3-4 kg over the first 6 months of TZD treatment [25], and the rate of weight gain decreases after the first 6–12 months [26]; however, in this work, no differences between treatments in DWG nor weight at slaughter was observed; this could be due to differences between the weight of the animals and the time they were in the experiment; moreover with a higher population of bulls we probably would be able to see an increase in DWG. Concerning DM intake, no differences were found between treatments (*P* > 0.1); however, other authors have reported increases during peripartum and postpartum periods in dairy cows with the use of 2,4-TZD [14, 27].

### 5.2. Carcass Quality

Although our data did not show significant differences between treatments, this could relate in some manner to the breed's characteristics. Limousin cattle are known to be late maturing as compared with Angus and Hereford [28], which means that our bulls might have not reached an adequate weight. Differences in maturing weight have been related with carcass yield, meat quality, and marbling; moreover, Limousins are categorized as low producers, when it comes to fat deposition [10].

### 5.3. Cellular Synthesis and Size

The glucose-lowering action of TZD is attributed to its agonistic action on peroxisome proliferator-activated receptor gamma (PPAR*γ*), a nuclear receptor that is expressed predominantly in adipose tissue and that regulates adipogenesis. In muscle and liver, which are the quantitatively most important tissues for insulin-dependent glucose homeostasis, TZD-induced insulin sensitization appears to occur associated with PPAR*γ*-mediated changes in lipid handling and signal output from adipose tissue [29]. While such a fat-mediated mode of TZD action is undisputed, the evidence accumulates that the pharmacology of TZD could be driven not only by PPAR*γ* activation, but also by PPAR*γ*-independent and nongenomic effects on mitochondria [30]. In this work, the lower muscle synthesis shown in (C) compared with the greater size of (T) might be due to 2,4-TZD action-related insulin stimuli, resulting in muscular glucose and fatty acids uptake from blood; this could indicate that cells were in a positive metabolic state. Therefore, (T) maintained cellular synthesis, while cellular protein degradation was lower compared with (C), and this was reflected in a larger size. Redistributing adipose tissue to visceral organs by inducing adipogenesis of smaller lipid droplets, which are more insulin sensitive, is one of the main important effects of TZD [31]; in humans and rodents, TZD increases the SC adipocyte cell surface [32]. Cell synthesis in omentum and perirenal was different between treatments, being higher for (C). It is well known that visceral adipose tissue cells are larger in size because of the union of lipid compared with SC droplets. Explanation of a larger size in (T) could be due to 2,4-TZD action resulting in accumulation of lipid droplets. Even though cell size did not exhibit differences in omental tissue; in perirenal tissue, (T) was higher than (C), which could mean that certain expression factors, such as interleukin (IL)-6, resistin, and PPAR*γ* are higher in visceral fat compared with SC fat, in which the expression comprises adipsin, leptin, and adiponectin [33, 34].

### 5.4. 2,4-TZD Presence in Blood, Liver, and Muscle

2,4-TZD concentrations were measured in blood, liver, and muscle in order to verify the presence of residues. As mentioned previously, blood samples were collected at the 28-day intervals. There was no evidence of TZD in blood (T). In contrast with other studies, TZD is supposed to have a 3-4 h elimination period [35], which explains its absence if samples were taken at around >12 h after the last meal when 2,4-TZD was administered. Hepatic samples from (T) presented significant differences from those of (C) (Table 3). Liver is the main route in which TZD is metabolized by cytochrome P450 enzymes and isoenzymes (CYP2C8 and CYP2C9) [36]. Liver injuries in terms of toxicity due to TZD doses (or toxicity during TZD treatment) are uncertain; troglitazone has been withdrawn from the market due to the mitochondrial hepatotoxicity [37]. In addition, rosiglitazone is metabolized by liver, and 64% and 23% of metabolites are excreted by feces and urine, respectively [12].

Muscle samples did not show the presence of TZD nor did its metabolites, but further research is required on hepatic and muscular pharmacokinetics and its correlation with doses, administration, and animal species.

### 5.5. PPAR Expression

In liver, lower expression of PPAR in (T) is consistent with that reported in the literature [38, 39]. TZD is markedly effective in reducing liver fat content by 30%–50% and in sensitizing the liver to insulin. This reduces the amount of endo- and exogenous insulin required to inhibit hepatic glucose production [40]. Studies with murine hepatic tissue have reported low expression of PPAR*γ* when pioglitazone has been utilized in a chronic treatment [41], similar to our results. On the other hand, PPAR*α* expression in tissues with high beta-oxidation activity, such as hepatic tissue, is frequently found.

Studies of PPAR expression in tissues have proved that when there is the severe insulin resistance in the muscular tissue, this could result in abnormal absence of the PPAR transcription factor [42]. Our results showed that PPAR possesses higher expression in (T) than in (C) in muscle. In the case of PPAR*α* in muscle, there is an evidence that the myogenic differentiation implicated in the process of mitochondrial biogenesis is governed by PPAR and in the coactivator PGC1-*α* in which they mediate OXPHEN (oxidative phenotype), which is the capacity of the muscle's activity, substrate, and metabolism implied in fiber type and established during myogenesis, repair, or hypertrophy of muscle [43].

PPAR*δ* participates in the development, induction, and maintaining of type I fibers, suggesting the conversion of some muscle fibers from type II to oxidative fibers, by inducing coactivator PGC-1*α* expression in skeletal muscle. Moreover, expression of PPAR*δ* has been related with genes involved in the regulation of lipid and glucose metabolism in human skeletal muscle [44]. The increase of PPAR*δ* in (T) is in agreement with other studies, in which unsaturated fatty acids have increased coactivator PGC-1*α* in differentiated myotubes, and it could be possible that PPAR*δ* ligands in muscle could be fatty acids and some of their metabolites [45]. On the other hand, some studies propose that PGC-1*α* levels in murines are inversely correlated with IM fatty acid levels, but, in this case, the animal's metabolism varies among species [45].

In muscle, PPAR*γ*, as well as PPAR*δ*, has been related with adipogenesis and is thought to contribute to transdifferentiation into adipocyte-like cells [46]. In agreement with the higher PPAR*γ* expression in SC tissue, cell synthesis in the 2,4-TZD treatment was as expected [47]. Moreover, studies in Holstein transition cows suggested a higher expression of PPAR*γ* in adipose tissue biopsies performed on the final day of treatment with 2,4-TZD [48]. First, SC adipose cells are more sensitive to the effect of the dietary nutrients [49]. SC tissue is well known to possess better cell differentiation than omentum; therefore, it is more receptive to the effect of TZD than other tissues [1, 50]. Moreover, evaluations of different subcompartments of SC and abdominal adipose tissues have observed posttranscriptional difference levels of adiponectin secretion and that the action of 2,4-TZD in SC fat could be related with abdominal adipose tissue changes [51]. It could be expected that animals that are not genetically predisposed to form marbling would deposit fat in SC rather than IM [52]. These could explain the effect of 2,4-TZD on (T) animals, concerning which we can discuss that SC depots were accumulating fat, and, probably, if we would increase the treatment length, we could have reached some marbling. In contrast, if we were to have used another breed, such as Angus, treatment with 2,4-TZD might have resulted in a larger amount of intramuscular fat. In addition, in our study, steers were also treated with trenbolone acetate and 17*β*-estradiol, since it has been proved that anabolic compounds increase protein synthesis, involving insulin-like growth factor (IGF)-I and decreasing protein degradation. On the other hand, anabolic compounds are known to promote reduction in body fat. Some studies have revealed that these compounds act by diminishing and blocking PPAR*γ* and C/EBP*α* [53]. Therefore, and according to our study's results, 2,4-TZD would contribute to the improvement of meat quality by its interaction with the anabolics to promote an increment in muscle and adipose tissue accretion.

## 6. Conclusions

The results show a positive effect of 2,4-TZD on muscle metabolism and PPAR*α* expression, which suggests increased fatty acid oxidation, thus the improvement in lipid metabolism. In adipose tissue, it appears that the effect of 2,4-TZD, combined with the anabolic used produced inhibition of PPAR*γ* expression in visceral adipose tissues (omentum and perirenal) according to cellular synthesis ratios. To our knowledge, this is the first study that considers the use of a TZD to improve beef production; further studies are required to provide information about dose, breed, and time of use, before its use of these in animal production could be considered.

## Figures and Tables

**Table 1:** Productive parameters in Limousin bulls with 2,4-Thiazolidinedione (TZD) (T) versus Control (C) animals.

Variables	Control	2,4-TZD
Initial weight (kg)	317 ± 45.5	308 ± 48.2
Final weight (kg)	517 ± 13.3	516 ± 11.8
Days at feedlot	136 ± 34.5	152 ± 41.8
Daily weight gain (DWG) (kg)	1.479 ± 0.2	1.407 ± 0.1
Liveweight (kg)	515 ± 13.9	514 ± 11.1
Carcass weight (kg)	324 ± 8.6	317 ± 9.2
Carcass yield (%)	63 ± 1.3	62 ± 1.3
Fat thickness (cm)	0.3 ± 0.2	0.4 ± 0.2
Longissimus dorsi muscle (LM) rib-eye area (cm^2^)	104 ± 29.4	114 ± 20.5

No differences were found (*P *> 0.05).

**Table 2:** Cellular synthesis and size in treated 2,4-Thiazolidinedione (TZD) (T), and Control (C) animals' tissues.

Tissues	Cellular synthesis RNA : DNA ratios			Cellular size DNA : protein ratios		
	C	T	*P *	C	T	*P *
Muscle	20.5	5.8	*	0.012	0.019	*
Adipose (omentum)	30.9	11.6	***	0.016	0.017	NS
Adipose (perirenal)	32.5	18.5	*	0.011	0.016	*
Adipose (SC)	14.8	59.0	***	0.014	0.006	**

NS: not significant*; *C: Control; T: Thiazolidinedione-(TZD-) treated*; *SC: Subcutaneous. *P < 0.05; **P < 0.01; ***P < 0.001.

**Table 3:** Comparisons of chromatography residues in liver tissue.

Treatment	Concentration*	MSE**
Control (C)	Not detectable	2.64*E*−8
2,4-TZD (T)	8.48*E*−7	2.64*E*−8

*Liver concentration (2,4-Thiazolidinedione (TZD) mg/g tissue); **MSE: medium standard error.

## References

[1] Kang JG, Park CY, Ihm SH (2010). Mechanisms of adipose tissue redistribution with rosiglitazone treatment in various adipose depots. *Metabolism*.

[2] Aranda A, Pascual A (2001). Nuclear hormone receptors and gene expression. *Physiological Reviews*.

[3] Michalik L, Auwerx J, Berger JP (2006). International union of pharmacology. LXI. Peroxisome proliferator-activated receptors. *Pharmacological Reviews*.

[4] Yessoufou A, Wahli W (2010). Multifaceted roles of peroxisome proliferator-activated receptors (PPARs) at the cellular and whole organism levels. *Swiss Medical Weekly*.

[5] Torii SI, Kawada T, Matsuda K, Matsui T, Ishihara T, Yano H (1998). Thiazolidinedione induces the adipose differentiation of fibroblast-like cells resident within bovine skeletal muscle. *Cell Biology International*.

[6] Hausman GJ, Poulos SP, Pringle TD, Azain MJ (2008). The influence of thiazolidinediones on adipogenesis *in vitro* and *in vivo*: potential modifiers of intramuscular adipose tissue deposition in meat animals. *Journal of Animal Science*.

[8] Killinger KM, Calkins CR, Umberger WJ, Feuz DM, Eskridge KM (2004). Consumer visual preference and value for beef steaks differing in marbling level and color. *Journal of Animal Science*.

[9] Pitchford WS, Deland MPB, Siebert BD, Malau-Aduli AEO, Bottema CDK (2002). Genetic variation in fatness and fatty acid composition of crossbred cattle. *Journal of Animal Science*.

[10] Wheeler TL, Cundiff LV, Shackelford SD, Koohmaraie M (2005). Characterization of biological types of cattle (Cycle VII): carcass, yield, and longissimus palatability traits. *Journal of Animal Science*.

[11] CICUAE

[13] Kuda O, Stankova B, Tvrzicka E (2009). Prominent role of liver in elevated plasma palmitoleate levels in response to rosiglitazone in mice fed high-fat diet. *Journal of Physiology and Pharmacology*.

[14] Smith KL, Stebulis SE, Waldron MR, Overton TR (2007). Prepartum 2,4-thiazolidinedione alters metabolic dynamics and dry matter intake of dairy cows. *Journal of Dairy Science*.

[15] Norma Oficial Mexicana

[16] Norma Oficial Mexicana

[17] N.A.M.P. North American Meat Processors Association

[18] Garcia RA, Phillips JG (2009). Physical distribution and characteristics of meat and bonemeal protein. *Journal of the Science of Food and Agriculture*.

[19] Bradford MM (1976). A rapid and sensitive method for the quantitation of microgram quantities of protein utilizing the principle of protein dye binding. *Analytical Biochemistry*.

[20] Reynoso CR, Mora O, Nieves V, Shimada A, González De Mejía E (2004). *β*-Carotene and lutein in forage and bovine adipose tissue in two tropical regions of Mexico. *Animal Feed Science and Technology*.

[21] Sripalakit P, Neamhom P, Saraphanchotiwitthaya A (2006). High-performance liquid chromatographic method for the determination of pioglitazone in human plasma using ultraviolet detection and its application to a pharmacokinetic study. *Journal of Chromatography B*.

[22] Muxlow AM, Fowles S, Russell P (2001). Automated high-performance liquid chromatography method for the determination of rosiglitazone in human plasma. *Journal of Chromatography B*.

[23] He J, Hu YF, Duan LF (2007). Sensitive and selective liquid chromatography-mass spectrometry method for the quantification of rosiglitazone in human plasma. *Journal of Pharmaceutical and Biomedical Analysis*.

[24] SAS Institute and Inc SAS/STAT User’s guide.

[25] Rizos CV, Elisaf MS, Mikhailidis DP, Liberopoulos EN (2009). How safe is the use of thiazolidinediones in clinical practice?. *Expert Opinion on Drug Safety*.

[26] Wilding J (2006). Thiazolidinediones, insulin resistance and obesity: finding a balance. *International Journal of Clinical Practice*.

[27] Smith KL, Butler WR, Overton TR (2009). Effects of prepartum 2,4-thiazolidinedione on metabolism and performance in transition dairy cows. *Journal of Dairy Science*.

[28] Dervillé M, Patin S, Avon L (2009). Les races allaitantes. Limousine. *Races Bovines de France*.

[29] Kushibiki S, Hodate K, Shingu H (2001). Insulin resistance induced in dairy steers by tumor necrosis factor alpha is partially reversed by 2,4-thiazolidinedione. *Domestic Animal Endocrinology*.

[30] Tontonoz P, Spiegelman BM (2008). Fat and beyond: the diverse biology of PPAR*γ*. *Annual Review of Biochemistry*.

[31] Sharma AM, Staels B (2007). Peroxisome proliferator-activated receptor *γ* and adipose tissue—understanding obesity-related changes in regulation of lipid and glucose metabolism. *Journal of Clinical Endocrinology and Metabolism*.

[32] Koenen TB, Tack CJ, Kroese JM (2009). Pioglitazone treatment enlarges subcutaneous adipocytes in insulin-resistant patients. *Journal of Clinical Endocrinology and Metabolism*.

[33] Eguinoa P, Brocklehurst S, Arana A, Mendizabal JA, Vernon RG, Purroy A (2003). Lipogenic enzyme activities in different adipose depots of Pirenaican and Holstein bulls and heifers taking into account adipocyte size. *Journal of Animal Science*.

[34] Haugen F, Drevon CA (2007). The interplay between nutrients and the adipose tissue: plenary lecture. *Proceedings of the Nutrition Society*.

[35] Hausman GJ, Dodson MV, Ajuwon K (2009). The biology and regulation of preadipocytes and adipocytes in meat animals. *Journal of Animal Science*.

[36] Scheen AJ (2007). Pharmacokinetic interactions with thiazolidinediones. *Clinical Pharmacokinetics*.

[37] Julie NL, Julie IM, Kende AI, Wilson GL (2008). Mitochondrial dysfunction and delayed hepatotoxicity: another lesson from troglitazone. *Diabetologia*.

[38] Bedoucha M, Atzpodien E, Boelsterli UA (2001). Diabetic KKAy mice exhibit increased hepatic PPAR*γ*1 gene expression and develop hepatic steatosis upon chronic treatment with antidiabetic thiazolidinediones. *Journal of Hepatology*.

[39] Sugden MC, Zariwala MG, Holness MJ (2009). PPARs and the orchestration of metabolic fuel selection. *Pharmacological Research*.

[40] Yki-Järvinen H (2009). Thiazolidinediones and the liver in humans. *Current Opinion in Lipidology*.

[41] Wierzbicki M, Chabowski A, Zendzian-Piotrowska M, Gorski J (2009). Differential effects of in vivo PPAR *α* and *γ* activation on fatty acid transport proteins expression and lipid content in rat liver. *Journal of Physiology and Pharmacology*.

[42] Hevener AL, He W, Barak Y (2003). Muscle-specific Pparg deletion causes insulin resistance. *Nature Medicine*.

[43] Remels AHV, Langen RCJ, Schrauwen P, Schaart G, Schols AMWJ, Gosker HR (2010). Regulation of mitochondrial biogenesis during myogenesis. *Molecular and Cellular Endocrinology*.

[44] De Lange P, Lombardi A, Silvestri E, Goglia F, Lanni A, Moreno M (2008). Peroxisome proliferator-activated receptor delta: a conserved director of lipid homeostasis through regulation of the oxidative capacity of muscle. *PPAR Research*.

[45] Schuler M, Ali F, Chambon C (2006). PGC1*α* expression is controlled in skeletal muscles by PPAR*β*, whose ablation results in fiber-type switching, obesity, and type 2 diabetes. *Cell Metabolism*.

[46] Holst D, Luquet S, Kristiansen K, Grimaldi PA (2003). Roles of peroxisome proliferator-activated receptors delta and gamma in myoblast transdifferentiation. *Experimental Cell Research*.

[47] Schoenberg KM, Perfield KL, Farney JK, Bradford BJ, Boisclair YR, Overton TR (2011). Effects of prepartum 2,4-thiazolidinedione on insulin sensitivity, plasma concentrations of tumor necrosis factor alpha and leptin, and adipose tissue gene expression. *Journal of Dairy Science*.

[48] Schoenberg KM, Overton TR (2011). Effects of plane of nutrition and 2,4-thiazolidinedione on insulin responses and adipose tissue gene expression in dairy cattle during late gestation. *Journal of Dairy Science*.

[49] Baldwin RL, McLeod KR, McNamara JP, Elsasser TH, Baumann RG (2007). Influence of abomasal carbohydrates on subcutaneous, omental, and mesenteric adipose lipogenic and lipolytic rates in growing beef steers. *Journal of Animal Science*.

[50] Soret B, Lee HJ, Finley E, Lee SC, Vernon RG (1999). Regulation of differentiation of sheep subcutaneous and abdominal preadipocytes in culture. *Journal of Endocrinology*.

[51] Walker GE, Verti B, Marzullo P (2007). Deep subcutaneous adipose tissue: a distinct abdominal adipose depot. *Obesity*.

[52] Harper GS, Pethick DW, Jones N The physiology of marbling: what is it, and why does it develop?.

[53] Johnson BJ, Chung KY (2007). Alterations in the physiology of growth of cattle with growth-enhancing compounds. *Veterinary Clinics of North America*.

